# Thymic origins of autoimmunity—lessons from inborn errors of immunity

**DOI:** 10.1007/s00281-020-00835-8

**Published:** 2021-02-02

**Authors:** Rosa Bacchetta, Kenneth Weinberg

**Affiliations:** 1 Division of Hematology, Oncology, Stem Cell Transplantation and Regenerative Medicine, Department of Pediatrics, Stanford University School of Medicine, Lokey Stem Cell Research Building 265 Campus Drive, West Stanford, CA 94305 USA; 2 Center for Definitive and Curative Medicine, Stanford University School of Medicine, Stanford, CA USA

**Keywords:** Thymic Regulatory T cells (tTreg), Medullary thymic epithelial cell (mTEC), Forkhead box P3 (FOXP3), Autoimmune regulator (AIRE), Primary immune deficiencies (PID), Primary immune regulatory diseases (PIRD)

## Abstract

During their intrathymic development, nascent T cells are empowered to protect against pathogens and to be operative for a life-long acceptance of self. While autoreactive effector T (Teff) cell progenitors are eliminated by clonal deletion, the intrathymic mechanisms by which thymic regulatory T cell (tTreg) progenitors maintain specificity for self-antigens but escape deletion to exert their regulatory functions are less well understood. Both tTreg and Teff development and selection result from finely coordinated interactions between their clonotypic T cell receptors (TCR) and peptide/MHC complexes expressed by antigen-presenting cells, such as thymic epithelial cells and thymic dendritic cells. tTreg function is dependent on expression of the FOXP3 transcription factor, and induction of *FOXP3* gene expression by tTreg occurs during their thymic development, particularly within the thymic medulla. While initial expression of *FOXP3* is downstream of TCR activation, constitutive expression is fixed by interactions with various transcription factors that are regulated by other extracellular signals like TCR and cytokines, leading to epigenetic modification of the *FOXP3* gene. Most of the understanding of the molecular events underlying tTreg generation is based on studies of murine models, whereas gaining similar insight in the human system has been very challenging. In this review, we will elucidate how inborn errors of immunity illuminate the critical non-redundant roles of certain molecules during tTreg development, shedding light on how their abnormal development and function cause well-defined diseases that manifest with autoimmunity alone or are associated with states of immune deficiency and autoinflammation.

## Introduction

Regulatory T cells (Treg) represent a specific T cell subset exercising a primary role in multiple essential immune functions. They regulate immune responses to self and foreign antigens, and control inflammation and immune surveillance. Regulatory T cells suppress the activation, expansion, and effector functions of the T cells that mediate immune responses following pathogen or alloantigen recognition. There are several types of regulatory T cells but only the naturally occurring CD4+ CD25hi FOXP3+ thymic Treg (tTreg) differentiate in the thymus as part of T cell ontogeny [1–4]. Induced Treg (iTreg) can be generated from CD4+CD25− cells in vitro in both mouse and human in the presence of TGFβ; however, human iTreg are not suppressive, suggesting that mouse and human Treg have different differentiation requirements [5]. FOXP3 is a master transcriptional regulator that controls the tTreg differentiation program, is essential for tTreg function, and its constitutive expression distinguishes tTreg from other cells, e.g., activated Teff, that transiently express FOXP3. A subset of Treg with a comparable phenotype (CD4+ CD25hi FOXP3+) has also been demonstrated in the mouse to differentiate in peripheral (i.e., extrathymic) tissues and is hence referred to as peripheral Treg (pTreg). Whether pTreg are also present in humans is uncertain since their identification is difficult because definitive markers that distinguish between the two subsets are presently missing [6]. Type 1 regulatory T cells (Tr1) are another peripherally induced CD4+ Treg population, which, in notable contrast to tTreg, develops and exerts its suppressive functions independent of FOXP3. Tr1 cells are also involved in maintaining immune tolerance and the balance between effector and suppressive activities, as recently reviewed elsewhere [7]. The occurrence of multi-organ autoimmunity, early in life, in patients with FOXP3 mutations demonstrate that tTreg are non-redundant, and that no other regulatory population, e.g., Tr1 or pTreg, can complement loss of FOXP3+ tTreg. This review will focus on tTreg and their early development, and will thus describe those intrathymic events that forge the cells’ unique identity under both physiological and prototypical disease conditions.

Since first described in the late 1990s in mice, a consistent body of knowledge has not only characterized the identity of tTreg but also described many, if not all, of the mechanisms by which these cells suppress Teff functions [8]. The precise molecular mechanisms that dictate tTreg differentiation, control the correct spatio-temporal manifestations of this complex developmental process, and thus enable a divergence from Teff differentiation remain, however, still largely undefined. Gaining a detailed understanding of tTreg development has been made possible by studying murine models, whereas comparable and complementary investigations in humans have been significantly more difficult to attain [9]. Therefore, most of our understanding of human thymopoiesis and specifically the role of the thymic microenvironment in this process has been extrapolated from mice with either spontaneously occurring or engineered loss and gain of gene functions. These studies take advantage of the ability to study embryonic thymic development or to trace cell lineages, which are not easily possible in human studies. However, postnatal thymopoiesis and tTreg progenitors can be studied in thymic tissue obtained from infants, children, and adults who undergo cardiac surgery, either for the correction of congenital heart disease (CHD) or for a heart transplant where the surgical procedures dictate the removal of thymic lobes. Consistent with primary immune deficiencies being informative “experiments of nature” to gain further insight into the immune development and function, the study of thymic tissue from genetic disorders affecting tTreg development, e.g., the chromosome 22q11.2 deletion syndrome (22q11.2DS) (discussed below and elsewhere in this issue), can be used as disease models analogous to murine models with defined genetic abnormalities [10]. However, the ability to study fetal development in human model diseases is limited and genetic heterogeneity can create additional confounders that are more easily controlled in murine models. Another approach to studying human thymopoiesis is chimeric mouse models transplanted with human hematopoietic stem and progenitor cells (HSPC). These models rely on interactions of the human HSPC with either the native murine microenvironments, co-transplanted human microenvironmental cells (e.g., human marrow and/or thymus), or “humanized” murine microenvironments that have been genetically altered to express human genes relevant to study hematopoiesis and immune functions. To date, the robustness of these chimeric mouse models is still limited by species-specific differences in cytokine ligand-receptor pairs and incomplete humanization of the murine MHC so that T lymphocyte differentiation, including that of Tregs, does not fully mirror human thymopoiesis [11]. A third approach to probe human T cell development uses thymus organoids, which have been developed to study in vitro early events in human thymopoiesis. To date, these platforms have not been used to examine intrathymic tTreg differentiation, selection, and maturation [12, 13]. While strategies to either expand tTreg or convert Teff into tTreg-like cells are the focus of intense research on the use of Treg as a cellular therapy, these in vitro or in vivo protocols may not be related to the normal development of tTreg [14, 15].

In this review, we will summarize the current knowledge of human tTreg identity and function, the role of thymic mechanisms that determine tTreg output, and the molecular and cellular basis for genetic diseases of tTreg function that lead to early-onset autoimmunity. Insights gained from these rare genetic diseases can be extended to improve the understanding of more common autoimmune diseases.

## tTreg identity and mechanisms of function

The phenotypic discovery and functional characterization of murine CD4+ CD25hi tTreg by Sakaguchi et al. in 1995 was subsequently extended to human peripheral blood where a corresponding phenotype was found [16–19]. Human CD4+ CD25hi tTreg described originally were themselves anergic, yet able to suppress the proliferation of naïve and memory effector T cells. They could also be stimulated to proliferate in vitro under specific short-term culture conditions [17–19]. Human tTreg represent a small (3–8%) subpopulation among peripheral CD4+ T cells. The number of tTreg is increased early in life in parallel with the higher lymphocyte count observed in infants, although the proportion of tTreg within the lymphocyte compartment remains relatively constant into adulthood [20]. Analyses of human T cell differentiation from fetal vs adult HSPC in chimeric mice indicate an inherent bias of fetal progenitors towards a Treg fate [21]. As in the mouse, human tTreg can be identified immunophenotypically by a constitutive high-level expression of the α chain of the IL-2 receptor (IL2R, CD25), and the lack of alpha chain of the IL-7 receptor (CD127) expression [3]. CD25 expression alone is an unreliable marker of tTreg because it is also expressed by activated Teff cells, albeit at lower levels. Because most Teff also express CD127, the combination of high CD25 and absent CD127 expression can distinguish tTreg from activated CD4 T cells [22]. Other functionally important surface markers of human tTreg include the cytotoxic T lymphocyte antigen 4 (CTLA4) and the Lymphocyte Activation Gene-3 (LAG-3). Importantly, the vast majority of CD4+ CD25hi CD127− cells (> 80%) express the transcription factor FOXP3 [23].

The constitutively high expression of nuclear FOXP3 is not only a phenotypic marker of tTreg, but is also necessary to establish the cells’ regulatory activity [23]. FOXP3 is a member of the forkhead (Fkh)-winged helix family of transcription factors, encoding a protein with a nuclear localization signal, a zinc finger, and leucine zipper–containing region, and an Fkh domain, all of which are necessary for function [24, 25]. Alternative splicing of human FOXP3 can generate 4 different isoforms (full length, Δ2, Δ7, and Δ2 Δ7, lacking exon 2, exon 7, or both exons 2 and 7) [26–29]. The most abundantly expressed human FOXP3 isoforms are the full length and the Δ2. The exon 2 encodes for the proline-rich domain and exon 7 contains the leucine zipper domain of FOXP3. The splice variants lacking exon 2 appear to be at least partially functional and to protect from severe IPEX syndrome [26–30], whereas the isoform lacking exons 2 and 7 is unable to confer suppressive function [30, 31].

One mechanism by which FOXP3 operates as a master regulator of T cell function is through direct interactions with genes containing the Fkh consensus binding sequence, GTAAACA. This recognition requires both the zinc finger/leucine zipper region and Fkh protein domains, as mutations in either abrogate FOXP3 function and result in autoimmunity [25]. At least 700–1400 genes are directly bound by the FOXP3 protein. However, the number of genes directly recognized by FOXP3 is estimated to be less than 10% that of the number of genes whose expression is regulated by FOXP3 [32]. This difference is explained by either activation of secondarily regulated genes by FOXP3-bound transcription factor genes or interactions of FOXP3 with other proteins, e.g., epigenetic modifiers that alter gene expression [25, 32]. Interacting transcription factor genes include those encoding NFAT, RUNX1/AML1, IRF4, GATA3, REL, and RORγT [25]. A recent study has demonstrated that many murine tTreg-associated genes are controlled by non-tTreg-specific transcription factors like TCF1, expression of which is modulated by FOXP3 [33]. The intermediary proteins have intrinsic capacity to bind to FOXP3-regulated genes, but the formation of macromolecular complexes with FOXP3 either potentiates or possibly stabilizes the effects of these other transcription factors. Examples of FOXP3 protein–protein interactions are the recruitment of DNA- or histone-modifying enzymes that alter the epigenome of FOXP3 controlled sequences by changing DNA methylation or post-transcriptionally modifying histone methylation or acetylation [25]. For example, FOXP3-dependent expression of the Ikaros family member Eos leads to the epigenetic silencing of the *IL-2* gene in tTreg [24, 34]. The central observation, for which our mechanistic understanding remains incomplete, is that the combined effects of the direct and indirect gene activation and repression confer a stable regulatory phenotype on FOXP3-expressing tTreg.

## Mechanisms of tTreg-mediated immune regulation

Immune regulation by tTreg depends on their ability to suppress the activity of Teff [35]. Much of the suppressive activity depends on cell–cell contact, indicating the important role of tTreg surface proteins. The LAG-3 expressed by tTreg binds to HLA-DR, thereby inhibiting the T cell receptor (TCR)-mediated activation of CD4+ Teff [36]. The binding of CTLA4 to CD80 and CD86 on APC blocks their interactions with CD28 expressed by Teff and thereby suppresses co-stimulation [37]. CTLA4 also upregulates indoleamine 2,3-dioxygenase (IDO) expression by dendritic cells (DC), which starves the T cells by depletion of tryptophan [38]. Perforin-mediated cytolysis of target cells by tTreg has been described as another mechanism of suppression [39]. Resting tTreg cells are typically hypoproliferative but, like activated Teff, are able to undergo IL-2-driven expansion in vivo; this property has been used in vitro to generate cell products that have been used for therapeutic purposes [14, 19]. Although tTreg do not produce IL-2 themselves, their expression of the CD25 subunit of the IL2R confers high-affinity binding of IL-2. Consequently, tTreg have a proliferative advantage in IL-2 responsiveness over other T cell populations [40–42]. Besides selectively promoting the growth of tTreg in response to IL-2, the high-affinity IL-2 receptor formed on tTreg by their high-level CD25 expression also scavenges IL-2 from their local milieu, thereby depriving Teff of this major T cell growth factor. Reflecting their indispensable role in Treg biology, the experimental loss of function of prototypical tTreg markers like CTLA4 or CD25 causes loss of tTreg function in mice, resulting in lymphoproliferative diseases accompanied by various degrees of autoimmunity [9]. In addition to IL-2 scavenging, there are other suppressive functions imposed by tTreg that are not cell contact dependent. For example, tTregs express the ectoenzymes CD39 and CD73, which generate immunosuppressive purine nucleosides [43]. The tTregs produce limited amount of cytokines, at least in vitro, and those include immunoregulatory cytokines such as IL-10, TGFβ, and IL-35, which can suppress some Teff activities [6].

The relative balance of suppressive mechanisms used by individual tTreg may depend on target cells and tissue milieu. Mature tTreg can undergo further differentiation in the periphery or may modulate their gene expression profile in response to activation state, distinctive milieus, or interactions with different types of Teff [44]. Like Teff, tTreg can also be subdivided by activation state. Resting and activated tTreg can be distinguished by expression of the CD45RA isoform in resting naïve tTreg and lack of CD45RA on activated tTreg [45]. Markers such as LAG3, TIGIT, PD-1, GARP, and TNFRSF18 (GITR) are expressed by or upregulated by tTreg undergoing differentiation to an activated state where they exert maximal suppressive function. These proteins are also found on peripherally induced pTreg [46]. Peripheral trafficking of tTreg into different tissues and continued encounter with multiple environmental factors further increase the cells’ heterogeneity [46]. Interestingly, different types of tTreg have been described in different tissues and in response to Th1, Th2, and Th17 subsets of Teff, suggesting that tTreg either adapt to their local milieu, or that specific tTreg subsets selectively traffic to or are selected by individual peripheral microenvironments [6, 47]. The ability to fully characterize human tTreg subsets and draw their differentiation trajectory remains limited in humans, although single-cell analyses are likely to at least partly redress these problems [48].

## Regulation of FOXP3 expression

Given the central role of FOXP3 in tTreg differentiation and function, understanding the mechanisms that regulate its expression has been critical for understanding tTreg biology. The *FOXP3* gene promoter is relatively weak, but is activated by binding to NFAT, AP-1, and the cyclic AMP-response element-binding protein (CREB)–activating protein 1 (ATF1), which are all downstream of TCR signaling, as well as two different forkhead family proteins, FOXO1 and FOXO3, which are downstream of CD28 co-stimulation and other pathways [25, 49, 50]. tTreg have a metabolic profile distinct from that of Teff, in which they use fatty acid oxidation (FAO) and pyruvate-dependent oxidative phosphorylation (OXPHOS) as their energy source [51]. By-products of these pathways, such as phosphoenol pyruvate (PEP) and mitochondrial reactive oxygen species (ROS), are predicted to increase NFAT activation after TCR signaling, which could increase *FOXP3* expression [51].

Because of the weak promoter, high-level *FOXP3* expression depends on three conserved non-coding sequences (CNS) that function as intronically located enhancer elements. CNS1 and CNS2 are in the first intron between non-coding exons -2b and -1, and CNS3 is in the second intron between coding exons 1 and 2 of the murine and human *FOXP3* genes [24, 25]. The distal CNS3 enhancer contains response elements for the NF-κB REL protein, which binds after TCR-mediated NF-κB activation. Together, the activation of the *FOXP3* promoter and CNS3 by TCR signaling to initiate *FOXP3* expression explain the expression of FOXP3 by activated Teff. Although TCR signaling alone is sufficient to induce *FOXP3* expression in mature human Teff, this is transient, as constitutive expression requires the tTreg-specific or pTreg-specific activities of CNS2 and CNS1, respectively. The transient expression of *FOXP3* by activated Teff, along with their activation induced CD25 expression, can lead to immunophenotypic confusion with tTreg, which constitutively express both FOXP3 and CD25. It is not known whether transient expression of FOXP3 by Teff is functional, e.g., putting a brake on Teff expansion. Phenotypically, tTreg can still be distinguished from activated Teff by their higher levels of CD25 expression and absence of CD127, as detailed above. CNS1, the most promoter proximal enhancer element, is important in the development of extrathymic pTreg. In these cells, *FOXP3* expression is induced via transcriptional activation resulting from binding of the TGFβ-related signaling protein SMAD3, NFAT, and retinoic acid receptor-retinoid X receptor (RAR-RXR) [25].

Stable *FOXP3* gene expression in tTreg is enabled epigenetically by the demethylation of a specific CpG island in CNS2 [52, 53]. Also known as Treg-specific demethylated region (TSDR), its epigenetic modification occurs exclusively, at least in humans, during tTreg differentiation, stabilizes the tTreg’s functional identity, and is maintained throughout the cell’s life, thus serving as a suitable molecular landmark. The demethylation of CNS2 allows binding by a number of transcription factors, including the aforementioned REL and CREB-ATF1, as well as RUNX1-core-binding factor subunit-β (CBFβ), ETS1, STAT5, and FOXP3 itself [25]. Since STAT5 is activated by IL2R signaling, the high-level expression of the CD25 subunit, along with the positive transcriptional effects of FOXP3 itself, generate feed-forward mechanisms for continued *FOXP3* gene expression. Further stabilization of *FOXP3* expression is provided by the recruitment of TET family demethylases by STAT5, thereby ensuring continuation of the demethylated state and accessibility of CNS2 to the transcription factors [54–56]. The protein–protein interactions, the necessity vs redundancy, and the temporal sequence of binding of the transcription factors that control *FOXP3* expression in human tTreg are not well characterized, but the overall effect is to stabilize *FOXP3* expression regardless of the cellular environment or activation state. The tTreg-specific demethylation of CNS2 is a unique epigenetic mark that allows for precise and unbiased quantification of tTreg among peripheral blood lymphocytes [57].

Several critical immunoregulatory pathways contribute to the maintenance of the tTreg phenotype by promoting *FOXP3* expression. Besides the growth advantage conferred by the IL-2R, its downstream activation of STAT5 results in recruitment of TET-demethylating enzymes to the TSDR, thereby creating a positive feedback loop that can reinforce *FOXP3* expression [25, 54–56]. In addition to IL-2, IL15 has also been described as important for STAT5-mediated tTreg survival and proliferation in the thymus [58]. While *FOXP3* expression by tTreg is generally stable, the pro-inflammatory cytokine IL-6 induces recruitment of DNMT1 methyltransferase to both the *FOXP3* promoter and the TSDR, which can cause remethylation of the embedded CpG island and repression of *FOXP3* expression [25, 54, 55]. This phenomenon is likely to contribute to the disruption of immune tolerance induced by IL-6.

## Intrathymic tTreg development: a pathway divergent from that of Teff cells

Given the importance of TCR and cytokine (IL-2) signaling for tTreg function in prevention of autoimmunity and maintenance of immune homeostasis, understanding the intrathymic events underlying the formation of their TCR repertoire and acquisition of signaling competence has been a subject of great research interest. Experiments to elucidate this important process derive mainly from murine models, whereas the precise cellular conditions and molecular events required to assure normal human tTreg development remain still ill-defined [3, 9]. Besides expressing unique sets of genes critical to their suppressive functions, human tTreg have a defined TCR repertoire which, in healthy subjects, is distinct from that of the Teff cells, in line with what has been observed in mice [58]. However, in one study of human Tregs, the overlap between the two TCR repertoires is higher than that reported in the mouse, resulting in greater heterogeneity of the human tTreg repertoire than suppositionally predicted if they were only reactive to self-antigens [59].

Similar to the process for Teff development, the engagement of clonotypic TCR with the self-peptide/MHC complex, at the double positive (DP) and early CD4 single-positive (SP) stages, both drives further differentiation and expansion and acts as a selecting signal for intrathymic Treg development. Initial positive selection of DP thymocytes in the thymic cortex depends on expression of TCR with adequately high affinity for a peptide/MHC complex. Thymocytes with high-affinity TCR for self-ligands ectopically expressed in the thymic medulla are subsequently deleted there, those with low affinity become naïve Teff cells, and T cells that recognize self-antigens via an intermediate affinity TCR develop into tTreg cells [60]. In the mouse, clonal deletion also occurs in the thymic cortex, but this has not been confirmed in the human thymus [61]. In contrast to the mouse where intrathymic tTreg commitment is first observed in the single-positive population, human CD25+ cells with demethylation of CNS2 (see below) and a tTreg-like TCR repertoire have been noted among DP cortical thymocytes. This earlier tTreg commitment in human thymus could be explained if TCR activation, e.g., through the *FOXP3* promoter and CNS3, is sufficient to initiate sustained FoxP3 expression [62].

Experiments in Nur77-GFP transgenic reporter mice have shown that Nur77-driven GFP expression is proportional to the TCR signaling strength. CD25+ tTreg progenitors had a significantly higher expression of Nur77-GFP than conventional CD4+ Foxp3− T cells, consistent with a higher degree of self-reactivity of the TCR expressed by tTreg cells. These findings confirm the importance of the TCR-peptide/MHC complex interactions in tTreg selection [63, 64]. Because the deletion of autoreactive Teff is leaky, some self-reactive T cells are present in the periphery [65, 66]. The experiments showing greater TCR signal strength in tTreg progenitors predicts that mature tTreg are ready to recognize self-antigens with a relatively higher affinity compared to Teff cells specific for the same self-antigens.

The signaling processes observed in mature tTreg after TCR engagement are thought to be similar to those induced in developing thymocytes. Activation of NF-κB and resultant Rel binding to CNS3 and NFAT binding to the promoter of the *FOXP3* gene are expected to lead to *FOXP3* expression and subsequent induction of other FOXP3-dependent genes, e.g., *CD25* and *CTLA4* [25]. However, TCR signaling is also predicted to cause transient FOXP3 expression in non-tTreg. Hence, this signal does not explain the chromatin remodeling and epigenetic modifications, including demethylation, occurring at CNS2 (TSDR) to assure constitutive FOXP3 expression. Crucially, the identification of FOXP3+ thymocytes does not establish that all such cells are tTreg progenitors. The establishment of constitutive *FOXP3* expression specifically in tTreg progenitors must depend on other processes besides TCR signaling. Interestingly, FOXP3 itself may alter TCR repertoire selection by tTreg progenitors. Using humanized mice transplanted with CD34+ hematopoietic stem cell progenitors in which *FOXP3* expression was persistently downmodulated with an siRNA modification, we observed direct control of the strength of TCR signaling by FOXP3, which correlated with Nur77 expression [67]. This direct effect would further support the importance of persistent high expression of FOXP3 in tTreg development and selection of a self-reactive TCR repertoire. The altered signal strength could only alter selection of FOXP3+ tTreg progenitors in the thymic medulla, since positive selection of immature cortical thymocytes occurs prior to onset of FOXP3 expression. Consistent with this understanding is the observation that in *Foxp3*−/− mice, the TCR repertoire has a higher degree of overlap between tTreg and Teff, compared to that of wild-type tTreg and Teff [63].

Besides the importance of intrathymic TCR signaling in establishing the tTreg repertoire and initiating *FOXP3* expression, cytokine signaling also plays a central role. IL-2 provides the survival and STAT5-mediated differentiation signals to CD25+ FOXP3− Treg progenitors, after which *FOXP3* expression begins [68, 69]. IL-2-inducible phospho-STAT5 (pSTAT5) is also likely to be critical to *FOXP3* transactivation by direct binding to CNS2, as well as by recruiting TET proteins to demethylate CNS2, thereby generating the TSDR essential for the establishment of constitutive *FOXP3* expression [54–56]. Within the murine thymic medulla where both tTreg and Teff are undergoing thymocyte negative selection, the Teff cells are the source of IL-2, further emphasizing the interrelationship between cells from these two developmental pathways [70, 71]. The thymopoietic cytokine IL-7, which also induces pSTAT5, seems less important for tTreg development, especially since IL-7 production in the thymic medulla is not robust [72, 73]. The negative to low levels of expression of the CD127 subunit of the IL-7R by tTreg is consistent with these observations. Although experimentally established only in murine models, the importance of Teff-derived IL-2 for intrathymic tTreg development is likely to be true in humans as well.

An alternative developmental pathway comprised of a cd25- FoxP3low tTreg progenitor has also been described in mice. The conventional cd25+ and Foxp3low tTreg progenitors display distinct dynamics for the imprinting of the tTreg cell–specific epigenetic signatures and downstream gene expression [68, 69]. Both types of progenitors generate mature tTreg with equal efficiency, but with non-overlapping TCR repertoires, suggesting again the developmental importance of intrathymic TCR-mediated signaling [69]. A fraction of the Foxp3low tTreg progenitors were found in the thymic cortex as well as in the medulla, raising the interesting question of whether their TCR repertoire is shaped by reactivity with cortical antigens. Treg derived from the two separate tTreg progenitors acquire different antigen specificities and may therefore have different roles in immune regulation. tTreg derived from the cd25+ progenitors protect from experimental autoimmune encephalomyelitis while those derived from Foxp3low progenitors protect from colitis [69]. The relevance to human tTreg development of these provocative findings of two different intrathymic developmental pathways that generate functionally non-redundant tTreg populations in the periphery has not yet been established.

## The thymic medulla in tTreg development and selection

The development of tTreg depends on the unique processes for TCR engagement during thymic development. The presentation of self-antigens to SP thymocytes occurs in the thymic medulla, and critically depends on medullary thymic epithelial cells (mTEC), which promiscuously express tissue-restricted antigens (TRA) otherwise specific to different peripheral tissues [74]. This process of promiscuous gene expression (PGE) has been aptly and vividly described as casting an immunological self shadow within the thymus to permit selection of the T cell repertoire [75]. As noted above, the intensity of the TCR signal determines whether medullary thymocytes adopt a fate of either a Teff or tTreg or, even undergo deletion, after exposure to TRA expressed by the mTEC.

The promiscuous expression in mTEC of thousands of genes encoding TRAs is driven by the transcription factor AIRE [75–77]. Adoptive transfer of T cells from *Aire*-deficient knockout mice into wild-type, T cell–depleted recipients resulted in autoimmunity [75]. However, unlike *Foxp3* mutations, the T lymphocyte defect is not intrinsic to lymphoid progenitors, as hematopoietic progenitors from normal marrow donors transplanted into *Aire*-deficient recipients still resulted in autoimmunity, thus establishing that the defective thymic microenvironment, and the absence of Aire expression in mTEC, which underlies the autoimmune phenotype. The expression of TRA by Aire-expressing mTEC was required for intrathymic deletion of autoreactive conventional T cells with a high-affinity TCR for TRA-derived peptides. Although the repertoire of TRA collectively expressed by Aire+ mTEC is broad, individual mTEC only express a limited number of TRA at any given point in time [78, 79]. Single-cell analyses indicate intercellular variability and stochastic patterns of TRA expression **[**79, 80]. A stochastic mechanism makes it unlikely that the self-shadow cast by Aire+ mTEC remains comprehensive. More recent data from single-cell transcriptome profiling studies support observations that promiscuous gene expression of TRAs exhibits ordered co-expression, although the mechanisms underlying this instruction remain biologically indeterminate. Ordered co-expression and random spatial distribution of a diverse range of TRAs likely enhance their presentation and encounter with passing thymocytes, while maintaining mTEC identity [80].

The role of AIRE in control of autoimmunity was first established by its positional cloning as the molecular cause of a complex pathology observed in patients diagnosed with autoimmune polyglandular syndrome 1 (APS1; a.k.a. the autoimmune polyendocrinopathy candidiasis ectodermal dystrophy (APECED) syndrome; discussed below). This causal link was subsequently confirmed in *Aire* knockout mice where disease penetrance was however shown to be dependent on other genetic influences [75**–**77]. The *AIRE* gene, located on chromosome 21q22.3, encodes a protein with domains that mediate oligomerization with other proteins relevant for its function [79] and assist in nuclear sublocalization to super-enhancer regions of DNA [80–82]. Promiscuous expression of TRA is promoted by AIRE binding and inhibition of molecules that normally repress gene expression. These targets include a repressive complex of ATF7ip (activating transcription factor 7-interacting protein) and MBD1 (methyl CpG-binding protein), and hypomethylated lysine 4 of histone H3 (H3K4me0) [83–88]. The mechanism and structural basis for transactivation of TRA by AIRE are still under active investigation.

AIRE and the thymic medulla are also important for generation of Tregs during thymocyte selection [89–92]. The murine models indicate that T cell progenitors with intermediate affinity for the TRA expressed by Aire+ mTEC are selected as Treg, and Aire deficiency causes reduction in tTreg number [2, 9]. Aire expression may also redirect T cells from a conventional T cell to a Treg fate because identical TCR specificities were found both on T cells infiltrating autoimmune targets in *Aire*-deficient mice and Tregs in wild-type animals [9, 93]. Similarly, self-reactive TCR sequences expected to be confined to Treg cells in healthy subjects are instead found as part of the conventional T cell TCR repertoire in APS1 patients, indicating altered Treg differentiation as a consequence of *AIRE* deficiency [94]. Interactions and transfer of AIRE-dependent TRAs from mTEC to thymic dendritic cells (DC) are an additional and essential mechanism to secure tTreg differentiation and thus self-tolerance [95–98]. The necessity of antigen transfer from AIRE-expressing mTEC to DC emphasizes the likely importance of medullary architecture in TCR repertoire selection. Extrathymic AIRE-expressing cells (eTAC) have been found in peripheral lymphoid tissues of both mice and humans. These eTAC can delete autoreactive T cells in the periphery, possibly via the expression of a restricted repertoire of TRAs, but their ability to induce regulatory T cells has not been established [99, 100].

Not all TRA expression by mTEC is AIRE dependent. There is evidence in mice that another transcription factor, Fezf2, may induce Aire-independent promiscuous expression of TRA. Analyses of *Fezf2*-deficient mice demonstrate that different patterns of autoantibodies and autoimmune targets are detected when compared to *Aire*-deficient animals [101]. Because genes controlled by either Aire or Fezf2 account for approximately 60% of all TRA loci, other molecular mechanisms must likely be in play to regulate the promiscuous transcription of the remaining loci. However, these are not yet known. Recently, the *Chd4* chromo helicase domain gene has been discovered as an upstream regulator of a smaller selection of both *Aire-* and *Fezf2*-controlled TRA loci [102]. *Chd4*-deficient mice develop autoimmunity with T lymphoid infiltration of peripheral targets, including the salivary gland, kidney, lung, and liver.

## When something goes wrong: the emerging domain of monogenic autoimmunity

Since first proposed by Good, inborn errors of immunity can be viewed as “experiments of nature” which provide clear-cut information on the importance and function of certain genes [103]. In his formulation, these defects anticipated the knockout technologies that have powered genetic studies in laboratory mice and other experimental models. Recently, with the broadening of genome sequencing and engineering, discoveries of new disease causative genes and mutation-specific variants originally observed in humans and subsequently experimentally verified in mice have grown at an increased pace.

## Primary defects of the lymphoid lineage in the generation of tTregs

According to the last published classification of primary immune deficiencies, there are 9 different gene defects that manifest with autoimmunity due to impaired Treg cells, defined as Tregopathies [104–107]. In addition to the classical immune dysregulation, polyendocrinopathy, enteropathy, and X-linked (IPEX) syndrome due to *FOXP3* mutation, the best characterized are *CD25*, *CTLA4*, and *LRBA* deficiencies, that is the absence of molecules as highly relevant for tTreg development and function, as mentioned earlier in this review [108–110]. Other recognized Tregopathies relate to a number of other defects. Gain of function mutations of *STAT3*, the latent transcription factor downstream of many cytokine receptor families, including IL-10 and IL-23, impair *FOXP3* and *IL-17* regulation and consequently interfere with the Treg and Th17 balance [111]. Deficiencies in *STAT5b* expression result in Tregopathies as this transcription factor initiates the regulatory cascade of the IL-2-CD25-FOXP3 axis [111]. More recently, rare Tregopathies have been recognized to be caused by *BACH2* mutations, a transcription factor that stabilizes *FOXP3* expression and represses Teff differentiation. [112, 113]. BACH2 is highly expressed in resting tTreg to maintain their quiescence, and is downregulated by activation [114, 115]. Finally, defects in regular tTreg development and function have been observed in patients with mutations of the beta subunit of the IL-2 receptor (*CD122*) or *DEF6*, which is involved in CTLA4 membrane trafficking [116–118].

Here, we will describe in more detail the prototypic autoimmune diseases of tTreg associated with *FOXP3*, *CD25*, and *CTLA4* mutations. Importantly, mutations in other genes affecting early stages of T cell development may manifest both profound immune deficiency with infections and autoimmune symptoms. Examples of such immune deficiencies are mutations of the recombination activating genes *RAG1* and *RAG2* necessary for V(D)J recombination and TCR repertoire formation, or of the Wiskott-Aldrich syndrome protein (*WASP*) resulting in defective antigen recognition in Wiskott-Aldrich syndrome. [107, 119–121]. The Omenn syndrome, which is caused by incomplete V(D)J recombination and abnormal TCR repertoire formation due to hypomorphic *RAG1/2* mutations, constitutes an excellent example of impaired tolerance induction due to an oligoclonal TCR repertoire and the escape of autoreactive T cells from stringent negative selection [120]. In Omenn syndrome, the failure of thymocyte maturation results in abnormal crosstalk between the thymocytes, mTEC, and dendritic cells, and abnormal medullary architecture and function [120] Consequently, intrathymic selection is abnormal so self-reactive T cells are released and undergo clonal expansion in the periphery, inducing autoimmunity directed against the skin, GI tract, liver, and/or blood cells that resembles graft-versus-host disease. Importantly, altered TCR signaling strength in the presence of a normal capacity for V(D)J recombination also impairs Teff and tTreg differentiation and tolerance acquisition [122]. The increased genomic analyses of patients with autoimmunity are expected to lead to the discovery of new gene mutations that cause Treg abnormalities.

## FOXP3 deficiency

Immune dysregulation polyendocrinopathy enteropathy X-linked (IPEX) syndrome is a rare but highly instructive example of a monogenic autoimmune disease of tTreg differentiation or function [106]. The analysis of a naturally occurring *Foxp3* mutation in the *scurfy* mouse enabled several groups of investigators to identify IPEX to be the consequence of mutations in *FOXP3*, which is localized on the X chromosome [123]. The clinical phenotype of male *scurfy* mice matched the clinical presentation of 19 related males patients initially reported in 1982 by Powell and colleagues [124]. Subsequent studies in transgenic and genetically engineered mice established the link between *FOXP3* mutations, a lack of tTreg, CD4+ T cell hyperproliferation, and tissue infiltration and destruction (reviewed in ref. 125). In most IPEX patients, the clinical features manifest themselves very early in life as severe multi-organ autoimmunity. In affected children, IPEX frequently presents in the first month of life and almost always within the first year [126, 127]. However, cases have been reported where the disease became apparent in utero and caused prenatal death, which highlights the importance of appropriate tTreg function early in life [128]. Enteropathy with watery refractory diarrhea is the typical common symptom of IPEX and is either preceded or followed in at least one-half of cases by autoimmune type 1 diabetes (T1D). Skin diseases in the form of severe eczema or psoriasis and cytopenias constitute other common features of the disease. As a result of both the dysfunctional tTreg suppressive activity and the intrinsic inability of mutated FOXP3 to regulate the cell cycle, CD4+ Teff cells are hyperproliferative [129]. Teff cell expansion is skewed towards a Th2 phenotype with more IL17 and IL22 production than in wild-type T cells, an imbalance that probably contributes to the development of autoimmunity. A secondary imbalance in the T follicular helper subsets and an increase in autoreactive B cells producing autoantibodies have also been described [130–132]. A subset of tTreg, T follicular regulatory (Tfr) cells, are normally found in germinal centers where they suppress T follicular helper (Tfh) cells and B cells to prevent autoantibody production [96, 133–135]. Although not yet established, autoantibody production in IPEX patients is likely due to Tfr defects, as shown in AIRE-deficient patients [96]. Despite the lack of functional FOXP3, CD4+ CD25+ CD127lo T cells are still present in IPEX patients. Phenotypic tTreg-like cells were also observed in mice carrying a null *Foxp3* allele that was marked with GFP [136]. This murine model and the clinical findings indicate that expression of some phenotypic markers of tTreg is not FOXP3 dependent. However, *FOXP3* mutations impair tTreg function, as measured by in vitro inhibition of proliferation and cytokine production by Teff [132].

In addition to the many preserved Treg phenotypic markers, the TSDR of the mutant *FOXP3* locus in tTreg of IPEX patients is completely demethylated, as in healthy subjects’ tTreg. However, the frequency of tTreg quantified by TSDR demethylation in IPEX patients is higher than that of healthy subjects [131]. While the meaning of this observation remains to be clarified, the demethylation of the TSDR in IPEX indicates that some epigenetic modifications of the *FOXP3* enhancer occur independently of FOXP3 expression during thymic Treg development, probably as a result of the intrathymic TCR or cytokine receptor–mediated signals. The fact that most relevant molecules involved in Treg identity and function have preserved expression in *FOXP3*-mutated Tregs, but that the cells remain dysfunctional, is an unexplained paradox that deserves further study. Consistent with the observation that FOXP3 activity is mediated by non-tTreg-specific transcription factors like TCF1, expression of Treg-associated genes can occur even when *FOXP3* is mutated [33]. The *FOXP3*-mutated tTreg may contribute to the pathology in IPEX not only because they lack adequate suppressive activity, but also because their functional identity is unstable and they can acquire effector functions [137, 138]. This understanding is supported by enrichment of FOXP3+ T cells among the IL-17-producing cells and increased FOXP3+ CD161+ co-expressing cells in the IPEX gut mucosa (132 and unpublished observation).

Correlations between IPEX genotype and phenotype have not been consistent, and the reasons for this discrepancy remain so far unknown [127]. Unlike *Foxp3* knockout mice, most patients are not null for *FOXP3.* Instead, expression is either decreased or mutant isoforms are generated that retain some biological activity. The incomplete loss of FOXP3 function probably explains the later onset of symptoms in patients compared to that of mice. As an example of the clinical heterogeneity, the clinical presentations and severity of IPEX can differ between siblings with identical *FOXP3* mutations. While the heterogeneity could be explained by modifying genes, unknown epigenetic modifiers, or environmental triggers for autoimmunity, the relative contributions of these variables to the clinical phenotype of IPEX are not yet understood.

Treatment of IPEX patients can be particularly demanding because control of the severe autoimmunity often requires multiple immune suppressive drugs, in addition to supportive care for enteropathy or hormone replacement therapy, e.g., insulin for T1D. Of the different immune suppressive drugs, rapamycin is the most efficacious. Rapamycin suppresses Teff while sparing the Treg cells and can activate FOXP3-independent regulatory pathways to increase suppressive activity [139, 140]. The only curative therapy currently available to treat IPEX is allogeneic hematopoietic stem cell transplantation (HSCT), which replaces the patient’s immune system and allows for the normal development of donor-derived tTreg and Teff cells [126].

## CD25 deficiency

Loss-of-function mutations of genes required for suppressive activity also cause tTreg dysfunction and autoimmunity. The first of these were mutations of *CD25*, the IL-2Rα subunit, in patients with an IPEX-like illness inherited in an autosomal recessive manner [108]. Like IPEX, patients with *CD25* deficiency have severe, early-onset enteropathy and eczema. Other frequently observed autoimmune manifestations include alopecia, thyroiditis, and cytopenias. However, *CD25* deficiency results in greater immune deficiency, especially susceptibility to severe viral infections, than is observed in IPEX.

The phenotype of *CD25* deficiency is consistent with its role in formation of the high-affinity IL-2 receptor, which is essential for both tTreg and Teff functions. The mutations described so far in IPEX-like patients lead to abrogation of membrane CD25 expression [41, 108]. Consequently, both intrathymic tTreg progenitors and mature peripheral tTreg lose a major survival and differentiation signal. The rare tTreg that are detected still express FOXP3, CTLA4, and TIGIT, and have normal TSDR demethylation. Besides the reduced number, the CD25-tTreg are functionally abnormal because of loss of the critical IL-2 scavenging function, as well as decreased expression of IL-2-dependent genes [42]. Because the IL-2R is also required for Teff expansion in response to infectious stimuli, the loss of CD25 results in the immune deficiency and susceptibility to viral infection. The higher susceptibility for infections is due to impaired IL-2-dependent expansion of Teff cells needed to efficiently clear infections. The greater severity of the IPEX-like phenotype than the immune deficiency in the *CD25-*deficient patients suggests that there is less redundancy for CD25 in tTreg development and function than in Teff cells. Consistent with this hypothesis, polymorphisms in the *CD25* locus that may decrease levels of CD25 expression are associated with susceptibility to T1D, with no evidence of immune deficiency [141]. The co-existence of autoimmunity and higher susceptibility for infections constitute a particular challenge in care of patients with *CD25* deficiency. As for IPEX, the only available curative treatment for patients with *CD25* deficiency is allogeneic HSCT [142].

## CTLA4/LRBA deficiency

Expression of CTLA4 by tTreg is functionally important for the suppression of Teff via blocking and/or transendocytosis of the CD28 co-stimulatory receptor for CD80/CD86 expressed by APC. Thus, CTLA4 acts as a brake on Teff activation. High-level surface expression of CTLA4 depends on the Liposaccharide-responsive beige-like anchor (LRBA), a membrane-anchoring protein that promotes recirculation of membrane CTLA4 from cytoplasmic pools [143]. Heterozygosity for loss-of-function mutations of *CTLA4* and biallelic *LRBA* mutations each result in impaired tTreg function and syndromic autoimmunity, either CTLA4 haploinsufficiency with autoimmune infiltration (CHAI) or LRBA deficiency with autoantibodies, Treg defects, autoimmune infiltration, and enteropathy (LATAIE), respectively [110, 144–146] . As in IPEX and *CD25* deficiency, CHAI and LATAIE patients frequently present with enteropathy and cytopenias, along with and lymphadenopathy and hepatosplenomegaly due to lymphoproliferation. Pulmonary and neurological pathologies may also be observed. Some patients have also developed malignancies, including lymphoma, gastric cancer, and EBV-associated malignancies. A peculiarity of *CTLA4* deficiency is the incomplete penetrance, which has been reported in a large study to be 67% [147]. There is also variance in disease severity within members of the same family carrying the identical mutation, rendering prognostication difficult. Both CHAI and LATAIE diseases can benefit from targeted treatment with abatacept, a recombinant fusion protein of CTLA4- and Ig Fc, which is a soluble molecule that can replace the function of membrane bound CTLA4 [110, 148]. Alternatively, mTOR inhibitors like rapamycin can inhibit CD28-mediated activation of Teff cells and provide benefit to these patients. LRBA-deficient cells can be induced in vitro to express CTLA4 after inhibition of lysosomal degradation, e.g., by chloroquine treatment, suggesting another potential avenue for pharmacologic treatment. However, HSCT remains the only potentially curative treatment, and is appropriate, in severe cases [147, 149].

## Gene therapy to restore or repair the Treg subsets and re-establish immune regulation

The engineered replacement of defective genes has a great potential as a definitive treatment of PIRD due to tTreg defects. In contrast, pharmacologic immunomodulation, e.g., rapamycin to inhibit Teff, does not restore tTreg function and requires life-long treatment to control or prevent autoimmunity. HSCT from an allogeneic donor has high risks of treatment-related morbidity and mortality associated with the use of cytotoxic chemotherapy and graft-versus-host disease. Persistence of autoimmunity has been occasionally observed after HSCT, possibly because of survival of either autoimmune Teff, or slow or inadequate development of new donor-derived tTreg [126]. Preclinical testing in experimental in vitro and murine in vivo models have established both proof-of-concept and feasibility for gene therapy as a disease-specific approach to restore tTreg function in patients with inborn errors of *FOXP3* or *CD25*. Targeting of the normal gene to HSC holds the promise of life-long production of genetically corrected blood cells. Because expression of genes like *FOXP3*, *CD25*, *CTLA4*, or *LRBA* is precisely controlled, successful gene therapy depends on the presence of each gene’s regulatory elements to assure appropriate cell type–specific expression, inductive conditions, timing, and levels of expression for the repaired sequence.

Two different gene therapy approaches have been explored to correct *FOXP3* mutations in IPEX patients [129, 149, 150]. CRISPR/Cas9-based gene editing has been tested to insert a normal full-length *FOXP3* coding sequence into mutant HSPC or T cells in vitro, using the endogenous regulatory elements to assure normal expression [149]. Lentiviral-mediated addition of the *FOXP3* coding sequence, along with a cassette containing the putative regulatory elements, has also been shown to confer appropriate expression and restoration of tTreg functions [150]. Instead of targeting HSPC, lentiviral-mediated transfer of wild-type *FOXP3* gene into pathogenic Teff can convert these cells into functional Treg. This lentiviral approach has been successful in murine models and is now being developed for a clinical trial [150]. For *CD25* deficiency, an innovative virus-free CRISPR/Cas9 gene–editing approach has been shown to efficiently restore normal CD25 expression in mutant T cells [151]. Given the toxicities and incomplete normalization of immune function that have been observed with allogeneic HSCT, these novel gene therapy approaches offer a combination of decreased risk of treatment-related complications like GVHD and potential cure of the PIRD due to tTreg defects.

## Impaired tTreg development due to abnormal thymic microenvironment

A number of primary genetic as well as acquired diseases of the thymic microenvironment result in immune dysregulation, marked by imbalances in thymic output between Teff and tTreg. The primary genetic diseases include those in which the function of an ostensibly normal thymic structure is impaired and others in which the formation of the thymic microenvironment is abnormal. Two examples of functional and developmental thymic defects are, respectively, APS1 due to *AIRE* mutation and the chromosome 22q11.2 deletion syndrome (22q11.2DS).

## Autoimmune polyglandular syndrome 1

The *AIRE* gene and pathogenic mutations were identified by positional cloning of the gene responsible for APS1, which was originally called autoimmune polyendocrinopathy candidiasis ectodermal dysplasia (APECED) [76, 77]. As discussed elsewhere in this issue, the classical manifestations of APS1 were a triad of chronic mucocutaneous candidiasis (CMC), hypoparathyroidism, and adrenal insufficiency, but the clinical phenotype is expanding with improved detection of *AIRE* mutations. This is analogous to the situation with IPEX where broader availability of genetic testing for *FOXP3* mutations has led to an expanded range of clinical manifestations and disease progression than predicted with the classical definition. An intriguing difference observed between APS1 and the engineered mouse model of *Aire* deficiency is the observation that patients present with decreased peripheral numbers of tTreg, which is not seen in the *Aire*− mice, despite their tTreg developmental defect. In addition to the altered TCR repertoire of tTreg due to intrathymic defects in selection, the tTreg from APS1 patients have intrinsic defects of decreased FOXP3 expression and function [89].

Given the dual role of AIRE expression by mTEC in clonal deletion of autoreactive conventional T cells and the positive selection of tTreg or re-direction of some cells to tTreg differentiation, the relative contributions of each defect in APS1 is an open question. For example, if the aberrant negative selection was accompanied by normal tTreg generation, would autoimmunity still occur? In athymic *Nude* mice transplanted with both a normal and *Aire*-deficient thymus, autoimmunity still developed despite the presumed normal generation of Treg by one of the thymi [152]. It is not known if the relative tTreg output of the mouse thymus is the same as human, nor is it known if transplanted thymus is equally as efficient as a native organ. Nevertheless, if these murine results were also true of APS1 patients, they would have significant therapeutic implications. First, if there were a therapy that allowed normal tTreg generation (such as the transplantation of fresh or cultured tissue [153] or in the future a functionally competent yet artificial thymic microenvironment), then thymectomy to remove the native *AIRE*-deficient thymus would be necessary to prevent further production of autoreactive Teff cells. Second, the efficacy of the newly produced tTreg would likely be enhanced if ablative lymphocytotoxic therapy was first administered to eliminate autoreactive T cells that had already been made. Anticipated risks of immunoablation include infection susceptibility such as occurs after HSCT, or transient autoimmunity due to imbalances of homeostatic expansion of Teff and tTreg populations in the lymphopenic environment.

The discovery of other genes and pathways like *Fezf2* and *Chd4* that regulate mTEC development and TRA expression in murine models make it likely that other defects of intrathymic production of tTreg will be discovered in patients with autoimmunity or other forms of immune dysregulation [101, 102]. To date, a clinical manifestation of human *FEZF2* mutation has not yet been described. Since *Fezf2* deletion is lethal in knockout mice because of brain and behavioral abnormalities, deletion or complete loss-of-function mutants are likely to be nonviable, but hypomorphic variants that specifically interfere with mTEC functions might be found in patients with immune dysregulatory disorders.

## COPA syndrome

In 2015, a cohort of five families with an autosomal dominant syndrome of autoantibodies, arthritis, and interstitial lung disease were identified by whole exome sequencing as having predicted loss-of-function mutations in the *Coatamer protein complex alpha* (*C*OPA) subunit gene [154]. COPA is required for transport of proteins from the endoplasmic reticulum (ER) to the Golgi apparatus as well as retrograde transport of selected proteins from the Golgi back to the ER like those with dilysine tags. The mutations in the COPA gene involve a WD motif that result in decreased binding to dilysine motifs and an impairment of intracellular protein trafficking. The patients with the COPA syndrome have decreased frequencies of interferon gamma–secreting Th1 cells and increased IL-17A-secreting Th17 cells. The reported autoantibodies have been classified as antinuclear, anti-neutrophil (ANCA), and rheumatoid factor-like, but unlike APS1, anti-interferon or other anti-cytokine antibodies have not been observed [154, 155]. Mice with a knock-in of one of the mutations observed in the original kindreds phenocopied clinical manifestations like interstitial and lung disease, as well as T cell activation and dysfunction [156]. The mechanism of autoimmunity in the COPA-deficient mice is the defective selection of autoreactive conventional T cells and decreased production of Treg. One hypothesis to explain this abnormal selection is inadequate expression of self-antigens, hence a mechanism analogous to that operational in APS1. However, COPA-deficient mice also show an abnormal expression of stimulator of interferon genes (STING), suggesting that there may be an excess in interferon-related inflammation [157]. The effects of aberrant intrathymic inflammatory signaling on tTreg generation are as yet unknown. The COPA syndrome raises the exciting possibility that other monogenic causes of specific autoimmunity will be found. For APS1, COPA, and any other autoimmune syndromes due to abnormal selection of conventional Teff cells and Tregs, key questions remain about the nature of TRA expression by the respective mTEC, the autoantigens recognized by the peripheral T cell populations, and the resultant autoimmune manifestations.

## Defects of thymic ontogeny leading to abnormal tTreg production

There are human diseases, notably 22q11.2DS and CHARGE syndrome, discussed elsewhere in this issue, which cause failure of thymic organogenesis, T lymphopenia, and increased risk of autoimmunity [10, 158, 159]. The 22q11.2DS syndrome results from interstitial deletion of 1.5–3.0 Mb of DNA on one copy of the long arm of chromosome 22 encompassing approximately 30–100 genes, of which hemizygosity for two genes, *TBX1* and *CRKL*, causes thymic hypoplasia in murine models [160]. The majority of patients with non-SCID levels of T lymphopenia are at risk not only for opportunistic infection but also high risk of autoimmune or allergic diseases [10, 161, 162]. The most commonly observed forms of autoimmunity are juvenile rheumatoid arthritis, autoimmune cytopenias, and thyroiditis. There is also a high incidence of allergy in the form of atopic skin diseases, asthma, and food allergies [163]. Analyses of the peripheral tTreg populations in 22q11.2DS patients have demonstrated overall reduction in the number of phenotypic CD4+ CD25hi CD127low FOXP3+ Treg**,** but their suppressive function is preserved [164] and unpublished observation). However, another study found abnormal Treg function in both intrathymic Treg progenitors and peripheral Treg populations [165]. Transplantation of allogeneic normal thymic tissue has been shown to restore peripheral Treg numbers, consistent with the Treg defects in 22q11.2DS patients being secondary to the stromal defect [153, 166].

Analyses of the thymus in patients with 22q11.2 DS has shown smaller than normal-sized medullary regions, reduced numbers of mTEC, and crucially decreased numbers of AIRE+ mTEC [165]. The lack of AIRE(+) cells is compatible with blocks in differentiation that prevent the maturation of AIRE− mTEC into later AIRE+ stages. The overall structure of the medullary regions is disorganized, which has been compared to an immature thymus. While a differentiation block is an attractive explanation for the thymic pathologies observed in the 22q11.2DS, the tools for topological analyses are not well enough advanced to distinguish “immaturity” from pathological disorder, e.g., an abnormal structure due to aberrant gene expression that are not simply a block of mTEC differentiation. The selection of tTreg progenitors requires topologically complex interactions with mTEC and DC, so pathological disorganization of the thymic medulla in 22q11.2DS is likely to be at least one cause of the abnormal tTreg production.

Besides the thymic hypoplasia, hemizygosity for *DGCR8* in 22q11.2DS provides a link to the autoimmunity of APS1. AIRE regulates a specific set of microRNAs, which are aberrantly expressed in AIRE− murine and human mTEC [167]. Some of these microRNA regulate promiscuous gene expression, with miR29a being a specific regulator of AIRE-dependent genes. DGCR8 is a component of the microprocessor complex, which cleaves canonical miRNAs. Mice with TEC-specific deletion of DGCR8 develop thymic hypoplasia and decreased expression of *Aire* in their mTEC [168]. The hemizygosity for *DGCR8* in the 22q11.2DS thymus is not expected to result in the same profound loss as in the homozygous *Dgcr8* murine knockouts, but the expected decrease in DGCR8 expression and possibly microprocessor complex activity could contribute to defective *AIRE* expression and tTreg development.

## Relevance to acquired diseases of tTreg generation

Thymic dysfunction and/or hypoplasia associated with decreased Treg numbers and autoimmunity has also been noted for acquired pathologies. Approximately 45% of patients with thymic epithelial tumors (TET, “thymoma”) have evidence of autoimmunity. While initially thought to be restricted to myasthenia gravis, extensive characterization of TET patients has shown endocrine disorders, chronic mucocutaneous candidiasis (CMC), and cytopenias like those seen in APS1 [169]. A proposed mechanism is the ability of these tumors to support T cell development but an inability to either delete autoreactive cells or generate normal tTreg because of a lack of expression of AIRE in the tumors [170, 171]. In support of this hypothesis, the clinical autoimmune presentations and range of autoantigens detected in TET patients resembles APS1 [172]. However, these patients frequently develop myasthenia gravis with autoantibodies directed against the acetylcholine receptor, a phenotype rarely seen in APS1. There is both an overlap and differences in autoantibodies directed against interferons in APS-1 (interferon ω) and TET patients with myasthenia (interferon α’s), suggesting that there are subtle differences in the patterns of autoreactivity accounting for phenotypic differences [173]. The general hypothesis of perturbed T cell development could be extended to include lack of expression by the tumor cells of other thymic medullary genes required to properly shape the T cell repertoire, e.g., *COPA* or possibly *FEZF2* or *CHD4*, which were discussed above. These tumors have not yet been comprehensively compared to normal thymic tissue, and such analyses could reveal non-AIRE-dependent mechanisms that contribute to failure to delete autoreactive, conventional T cells or to promote the development of tTreg. Complicating the interpretation of the peripheral T cells is the co-existence of both TET and normal thymus tissue. The development of autoimmunity may reflect the inability of the normal thymic tissue to produce sufficient tTreg to compensate for the autoreactive conventional T cells aberrantly generated within the tumor. Surgical removal of the tumor frequently results in amelioration of the autoimmune symptoms, but many patients have persistent autoimmunity and may even require immunosuppressive therapy.

Another relevant condition in which thymic dysfunction is likely to contribute to abnormal T cell repertoire formation and autoimmunity is chronic graft-versus-host disease (cGVHD) occurring after allogeneic hematopoietic stem cell transplantation [174]. Acute GVHD (aGVHD) occurs when histoincompatible T lymphocytes engraft in an immunosuppressed transplant recipient, usually resulting in T cell–mediated inflammatory injury to targets of the skin, gastrointestinal tract, and/or liver. In contrast to aGVHD, there is fibrosis and frequent incidence of autoimmune diseases in cGVHD. The relationship between acute and chronic GVHD is complex: different pathologies (inflammation vs fibrosis, not all patients with aGVHD develop cGVHD, cGVHD can develop in patients with no prior evidence of clinical aGVHD, and alloreactivity vs autoimmunity). Patients with aGVHD have decreased levels of circulating Treg, while paradoxically, cGVHD patients have higher numbers of circulating Treg [175]. It has been long known that the thymic epithelium is injured in aGVHD, but the clinical implications difficult to study in humans [176, 177]. A pathophysiologic linkage between aGVHD and cGVHD has been provided by analyses of the immunologic effects of TEC damage caused by aGVHD in murine transplant recipients. TEC damage by alloreactive T cells results in loss of Aire+ mTEC and prevents the deletion of autoreactive cells [178, 179]. The effects on Treg populations were not studied, but based on the dual roles of the mTEC in deletion of autoreactive conventional thymocytes and promotion of tTreg development, it is reasonable to speculate that tTreg production is also impaired. Overall, the data from GVHD experiments indicate that analogously with APS1 or 22q11.2DS, secondary defects of mTEC number and function leading to abnormal tTreg generation are relevant to the acquired autoimmunity observed in HSCT recipients.

## Conclusions

Just as monogenic diseases causing clinical immune deficiency provided a window into how the immune system controls infection, the primary immune regulatory disorders are illuminating the mechanism by which autoimmunity is prevented. Among those, the diseases that interfere with tTreg development or function have established the importance of transcription factors like FOXP3 and AIRE, and the roles of the differentiation program of tTreg progenitors and the thymic medulla in tTreg development. The clinical entities of IPEX and APS1 caused by the mutations of *FOXP3* and *AIRE*, respectively, are likely to be the first of a string of new primary monogenic diseases that can be used for discovery of immune regulatory mechanisms and targeted interventions.
